# Photoreceptor Protection by Mesencephalic Astrocyte-Derived Neurotrophic Factor (MANF)

**DOI:** 10.1523/ENEURO.0109-18.2018

**Published:** 2018-04-20

**Authors:** Jianmin Lu, Lingyu Luo, Deqiang Huang, Xian Liu, Xin Xia, Zhengying Wang, Byron L. Lam, Jinglin Yi, Rong Wen, Yiwen Li

**Affiliations:** 1 Bascom Palmer Eye Institute, University of Miami, Miller School of Medicine, Miami, FL 33136; 2 Department of Ophthalmology, First Affiliated Hospital, Dalian Medical University, Dalian, 116011, China; 3 First Affiliated Hospital, Nanchang University, Jiangxi Medical School, Nanchang, 330006, China; 4 Eye Hospital, Nanchang University, Jiangxi Medical School, Nanchang, 330006, China

**Keywords:** degeneration, MANF, neuroprotection, photoreceptor, retina, RGC

## Abstract

Retinal degenerations are a major cause of vision impairment and blindness. Neuroprotective therapy is a promising therapeutic strategy for retinal degenerative diseases. We investigated a novel neurotrophic factor mesencephalic astrocyte-derived neurotrophic factor (MANF) in the retina. MANF is expressed at a high level during postnatal development and the expression declines to a lower level as the retina matures. Müller cells are the major cells expressing MANF. It is also found in the retinal ganglion cells, in the inner nuclear layer (INL) neurons, and in retinal pigment epithelial (RPE) cells. Intravitreal injection of recombinant human (rh)MANF significantly protected rod and cone photoreceptors in rats carrying the rhodopsin S334ter mutation, and preserved electroretinograms (ERGs) in the *rd10* (*Pde6b^rd10/rd10^*) mice. These results indicate that MANF is a native protein in the retina and is a potent neurotrophic factor for photoreceptor protection.

## Significance Statement

This is a study of high translational value to examine the neuroprotective potential of a novel neurotrophic factor mesencephalic astrocyte-derived neurotrophic factor (MANF) in the retina. MANF is expressed in the retina at high level during postnatal development and then declines as the retina matures. Recombinant MANF protects rod and cone photoreceptor cells and preserves electroretinograms (ERGs). These results suggest a role of MANF in the retinal development and provide preclinical evidence for further development of MANF as a neuroprotective agent as a potential treatment for retinal degenerative disorders.

## Introduction

Retinal degenerations are a major cause of vision impairment and blindness (Hartong et al., 2006). Retinitis pigmentosa (RP), a most common inherited retinal degeneration, affects one in 3500–4000 people. Mutations in more than at least 60 genes (as of February 2018) are identified to be associated to RP (RetNet, 2018). Yet in many RP cases, the causative mutations remain unidentified (Hartong et al., 2006).

There are no specific treatments available for retinal degenerations, except a recently approved gene therapy for *RPE65* related Leber’s congenital amaurosis type 2 (LCA2; Food and Drug Administration, 2017). Three major treatment strategies are under intensive research: gene therapy or gene augmentation therapy, neuroprotective therapy, and retinal prostheses. In gene therapy, a copy of transgene (cDNA in most cases) encoding a normal gene product is transferred to affected cells to restore the function of faulty gene, and thus the current therapy strategy is limited to the treatment of loss-of-function mutations in identified genes. The success of gene therapy for LCA2 proves that the function of *RPE65* gene can be restored by introducing the normal *RPE65* transgene into the retinal pigment epithelial (RPE) cells (Bainbridge et al., 2008; Hauswirth et al., 2008; Maguire et al., 2008). Surprisingly, the *RPE65* gene therapy failed to stop the degeneration process (Cideciyan et al., 2013; Bainbridge et al., 2015; Jacobson et al., 2015). Therefore, restoration of a defect gene may not necessarily protect photoreceptors from degeneration, and there is a need for additional treatments to save photoreceptors.

Retinal prostheses are electronic implants to restore vision and to improve quality of life for patients with end-stage retinal degenerations (Cheng et al., 2017). A retinal prosthesis uses an array of electrodes to stimulate the remaining retina, which in turn conveys the signals to the brain. Due to the large size and limited number of electrodes in an array, the quality of images experienced by patients is very low. Nevertheless, the limited visual function dramatically improves the quality of life of patients with total blindness.

Neuroprotective therapy aims at delaying or halting the degenerative process in neurons by neurotrophic agents. It is a “broad spectrum” strategy to save neurons that one neurotrophic agent may be effective for degenerations caused by more than one mutant gene. In addition, a neurotrophic agent effective for photoreceptor protection may also be effective for other retinal neurons. For example, ciliary neurotrophic factor (CNTF), the best studied neurotrophic factor for retinal degenerations (Wen et al., 2012), has been investigated for a variety of neural degenerative diseases in the retina, including RP (Sieving et al., 2006; Talcott et al., 2011; Birch et al., 2013, 2016), macular degeneration (Zhang et al., 2011), and macular telangiectasia (Chew et al., 2015; Sallo et al., 2018).

Mesencephalic astrocyte-derived neurotrophic factor (MANF) is a novel neurotrophic factor originally identified as a secreted protein in the culture medium of rat mesencephalic type 1 astrocytes (ventral mesencephalic cell line 1; VMCL1) that promotes survival of rat embryonic dopaminergic neurons (Petrova et al., 2003). It protects nigrostriatal dopaminergic neurons from 6-hydroxydopamine-induced degeneration *in vivo* (Voutilainen et al., 2009) and brain cells in a rat stroke model (Airavaara et al., 2009). In the present work, we characterized the expression of MANF in the retina. MANF is a retinal native protein, expressed in Müller cells, RPE cells, and neurons in the inner retina. When delivered by intravitreal injection, recombinant human MANF (rhMANF) significantly protects rod and cone photoreceptors from degeneration. These results indicate that MANF is a neurotrophic factor for photoreceptors and provide evidence to support the development of MANF for treating retinal degenerative diseases.

## Materials and Methods

### Expression and purification of rhMANF protein

**Figure 1.**
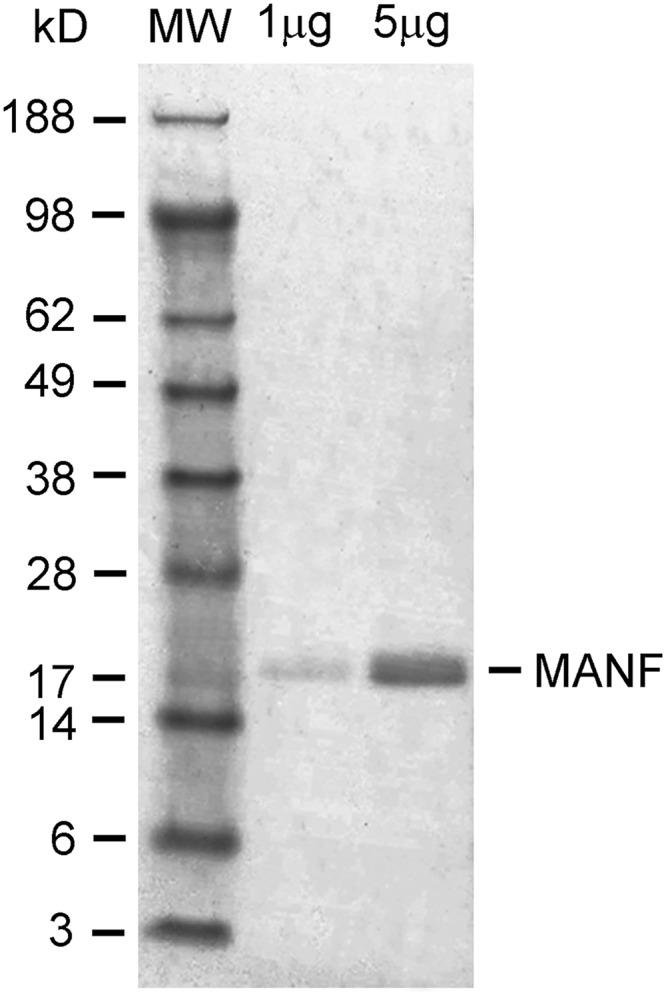
Expression and purification of rhMANF. The 6xhis-tagged protein was expressed in *E. coli* and purified on nickel columns. Purified rhMANF was detected as a single band ∼20 kDa when electrophoresed on 4–12% NuPAEG gel and visualized with Coomassie Blue.

Recombinant human MANF protein expression and purification were conducted as described previously (Wen et al., 2006). The open reading frame of mature human MANF cDNA was cloned into an expression vector pQE30 (QIAGEN), fused to a 6xHis tag at the amino terminus to generate plasmid pQE-MANF. MANF was expressed in *Escherichia coli* (XL-blue, Agilent) and purified by immobilized-metal affinity chromatography on Ni-NTA Agarose columns (QIAGEN) under native conditions. Protein was eluted from the Ni-NTA columns with a buffer containing 250 mM imidazole. The elution buffer was exchanged to phosphate buffered saline (PBS) on Econo-Pac 10DG columns (Bio-Rad Laboratories). The purified rhMANF in PBS was stored at -80°C until use. The protein has an apparent size of ∼20 kDa after electrophoresis on acrylamide gel (Fig. 1).

### Experimental animals

All procedures involving animals were approved by the Animal Care and Use Committee at the University of Miami. Transgenic rats (Sprague Dawley background) carrying a murine rhodopsin mutant S334ter (line3 or S334ter-3), wild-type Sprague Dawley (Harlan Laboratories) rats, and the *rd10* (*Pde6b^rd10/rd10^*) mice that carry a missense mutation in the *Pde6b* (cGMP phosphodiesterase 6B; The Jackson Laboratory), were kept in a 12/12 h light/dark cycle at an in-cage illumination of <50 lux. The room temperature was maintained at 20–22°C. Heterozygous S334ter-3 rats were produced by mating homozygous male breeders with wild-type Sprague Dawley females. Animals of both sexes were used in the experiments.

### Intravitreal injections

Intravitreal injections were delivered through 33-gauge needles connected to 10-µl microsyringes (Hamilton Company), as described previously (Wen et al., 2006). The right eye of an animal was injected with rhMANF protein and the left eye with the equivalent volume of PBS.

### Histology

Retinal structure was examined with semi-thin sections. Animals were killed by CO_2_ overdose, immediately followed by vascular perfusion with mixed aldehydes (LaVail and Battelle, 1975). Eyes were embedded in an Epon/Araldite mixture, sectioned at 1 µm thickness to display the entire retina along the vertical meridian (LaVail and Battelle, 1975). Retinal sections were stained with 1% toluidine blue and examined by light microscopy.

### Western blotting

To examine MANF expression levels by Western blot analysis, retinas were harvested after animals were killed by CO_2_ overdose. Pooled retinas were homogenized in a lysis buffer that contained 50 mM Tris, 150 mM NaCl, 1% NP-40, 0.1% sodium dodecyl sulfate, 20 nM calyculin A, 100 mM phenylmethylsulfonyl fluoride, 10 µg/ml leupeptin, and 10 ng/ml aprotinin. Protein concentration in each sample was determined by the BCA protein assay (Bio-Rad Laboratories). Total protein of samples was electrophoresed on 4–12% NuPage gel (ThermoFisher Scientific) and transferred to nitrocellulose membranes (Bio-Rad Laboratories). Western blotting was performed using rabbit anti-MANF antibodies (1:1000 dilution, MilliporeSigma) and mouse anti-β-actin antibodies (1:5000, dilution, MilliporeSigma), followed by appropriate secondary antibody conjugated to horseradish peroxidase (HRP). Signals were visualized using chemiluminescent substrates (ThermoFisher Scientific) and recorded on Hyperfilm (GE Healthcare). All experiments were repeated 3 times to verify the consistency of the results.

### Immunocytochemistry

Immunocytochemical experiments were performed on cryo-sections. Eyes were removed after animals were perfused with 4% paraformaldehyde. Eyecups were prepared, cryo-protected with 30% sucrose, and frozen in Tissue-Tek OCT compound (Miles Inc.). Cryo-sections of 12 µm were cut along the vertical meridian on a Cryostat (CM1900, Leica Biosystems), incubated with rabbit anti-MANF antibodies (1:400 dilution, MilliporeSigma), and with mouse anti-glutamine synthetase (GS; 1:100 dilution, MilliporeSigma) to identify Müller cells. MANF and GS immunoreactivities were visualized by Cy2- and Cy3-conjugated secondary antibodies (Jackson ImmunoResearch), respectively, and examined by confocal microscopy.

### Retina-lens preparations and peanut agglutinin (PNA) staining

Retina-lens preparations were used for PNA staining to identify cone outer segments (COSs), as described previously (Li et al., 2010). Eyes were removed from animals after CO_2_ overdose and cardiac perfusion with PBS. Retina-lens preparations were obtained by first removing the cornea along the limbus. A small cut was made at the edge of sclera toward the posterior pole. With two pairs of forceps placed one at each side of the cut, the sclera, along with the choroid and RPE was carefully torn open. The sclera-choroid-RPE was peered to the base of optic nerve, and cutoff. The resulted retina-lens preparations were post-fixed in 4% paraformaldehyde solution at 4°C for 4 h.

PNA, which binds to COSs (Blanks and Johnson, 1983; Hageman and Johnson, 1986; Li et al., 2010), was used to identify cone photoreceptors. For staining, retina-lens preparations were incubated with Alexa Fluor 488-conjugated PNA (ThermoFisher) for 1 h. The lenses were removed and the retinas were flat-mounted on slides and examined by confocal microscopy. Cone cells were counted in the superior retina.

### Electroretinogram (ERG) recording

ERGs were recorded with a UTAS system (LKC Technologies). After dark adapted overnight, mice were anesthetized with intraperitoneal ketamine (80 mg/kg) and xylazine (4 mg/kg) under dim red light. Pupils were dilated with 0.1% atropine and 0.1% phenylephrine HCl. During the recording, animals were on a heat pad to maintain body temperature at 37°C. A contact lens electrode was placed on the cornea of each eye, a differential electrode under the skin of the forehead, and a ground wire electrode under the skin close to the base of the tail. Both eyes were recorded simultaneously. Full field ERGs were elicited by 1-ms white flashes generated by white LEDs in the Ganzfeld sphere. Inter-stimulus intervals were 10 s. Each recording was an average of 10 responses. The b-wave amplitudes of the treated eyes were compared with the control eyes.

### Experimental design and statistic analysis

All means are presented as mean ± SD. A minimal number of animals to be used in each experiment was determined by our previous experience on similar experiments and power analysis based on α = 0.05 at 80% statistical power. Data analyses were performed using InStat (version 3.0, GraphPad Software Inc.). Results were evaluated by Student’s *t* test for comparisons between two experimental groups.

## Results

### Expression and localization of MANF protein in the retina

**Figure 2.**
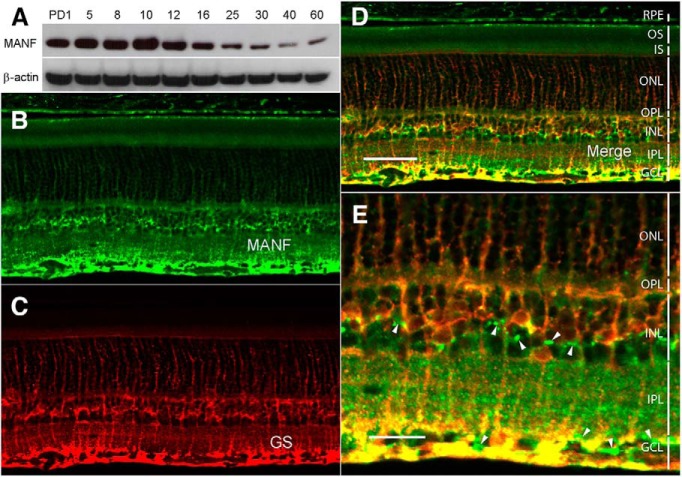
Expression of MANF in the retina. ***A***, Expression of MANF during postnatal development. Retinas were collected at designated time points from wild-type Sprague Dawley rats. Levels of MANF were detected by Western blot analysis with anti-MANF antibodies. High levels of MANF expression were detected during postnatal development (from PD1 to PD16; ***A***, upper panel). As the retinas mature, the expression decreases (from PD25 to PD60; ***A***, upper panel). The same blot was reprobed with anti-β-actin antibodies as loading controls (***A***, lower panel). Cryosections of 12 µm of eyes of PD20 wild-type Sprague Dawley rats along the vertical meridian were stained with anti-MANF antibodies (***B***, ***D***, ***E***). Müller cells were identified by antibodies against GS (***C–E***). Extensive colocalization of MANF and GS immunoreactivities (***D***, ***E***, yellow) indicates that MANF in the retina is expressed in Müller cells. RPE cells, neurons in the GCL, and neurons in the INL also display MANF immunoreactivities (***B***, ***D***, white arrowheads in ***E***). OS, outer segments; IS, inner segments; OPL, outer plexiform layer; IPL, inner plexiform layer. Scale bar: 50 µm (***D***) and 20 µm (***E***).

The expression of MANF protein was examined in the retinas of Spraque Dawley rat at postnatal day (PD)1, PD5, PD8, PD10, PD12, PD16, PD20, PD25, PD30, PD40, and PD60 by Western blot analysis. High level of MANF protein expression was detected in the rat retina from PD1 to PD16 with the highest level at PD10. The expression declines to lower levels from PD25 to PD60, as the retina matures (Fig. 2*A*).

MANF was localized in retinal cells by immunostaining in cells in the inner nuclear layer (INL), the retinal ganglion cell layer (GCL), and the RPE layer (Fig. 2*B*,*D*,*E*). Double staining of MANF and GS, a Müller cells marker, shows extensive colocalization of MANF and GS immunoreactivities, indicating most of the MANF in the INL is in Müller cells (Fig. 2*C*,*D*,*E*). MANF immunoreactivity was also detected in neurons in the INL and the GCL (Fig. 2*B*,*D*,*E*, white arrowheads) and in the RPE cells (Fig. 2*B*,*D*). Although MANF is a secreted protein, it contains an endoplasmic reticulum (ER) retention sequence RTDL at the C-terminal end to allow MANF retained in the ER in the cells that express it (Henderson et al., 2013). A certain amount of MANF should remain in MANF-expressing cells to be detected by immunocytochemical experiments. It is therefore very likely that MANF-positive cells are MANF-expressing cells.

### Protection of rod photoreceptor by MANF treatment

**Figure 3.**
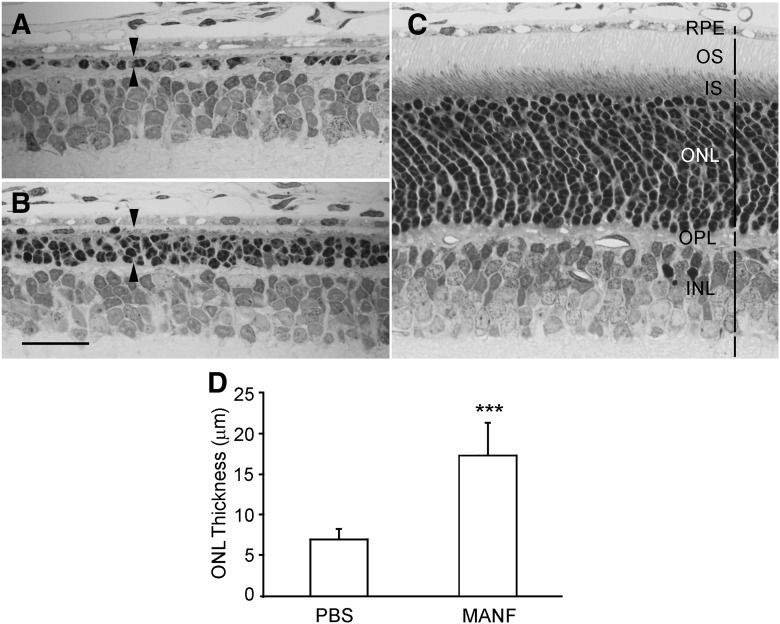
Protection of rod photoreceptors by MANF. The right eyes of S334ter-3 rats were treated with 6 µg (in 3 µl of PBS) of MANF by intravitreal injection, and the left eyes were injected with of PBS. Tissues were collected at PD20. Semi-thin sections were cut and stained with toluidine blue. The ONL in the control eye had only one row of nuclei in the superior retina (between arrowheads, ***A***), whereas in the eyes treated with MANF, there are three to four rows of nuclei in the ONL (between arrowheads, ***B***). In comparison, the ONL in a normal wild-type rat at PD20 contains 13–14 rows of nuclei (***C***). Quantitative analysis shows that the ONL in the MANF-treated retinas (superior retina) is significantly thicker than that in the PBS-treated retinas (***D***, triple asterisks indicate *p* < 0.001, Student’s *t* test). OS, outer segments; IS, inner segments; OPL, outer plexiform layer. Scale bar: 25 µm.

We examined the potential protective capability of MANF on photoreceptors in heterozygous S334ter-3 rats. Photoreceptors in those animals undergo rapid degenerate starting around PD10 and by PD20, most of the rod photoreceptors are lost (Liu et al., 1999). Rod outer segments fail to develop in the S334ter-3 rats, and the scotopic ERGs are undetectable. MANF was injected intravitreally to the right eyes of S334ter-3 rats (6 µg in 3 µl of PBS) at PD9, and the left eyes were injected with 3 µl of PBS as controls. Eyes were collected by PD20 for histologic analysis. As shown in Figure 3*A*, the outer nuclear layer (ONL) of the superior retina in the control eyes had only one row of nuclei. In the treated eyes, however, the ONL in the superior retina had three to four rows of cell nuclei (Fig. 3*B*). In comparison, the ONL in a normal PD20 rat has 13–14 rows of photoreceptor nuclei (Fig. 3*C*). Quantitative analysis of the ONL thickness, measured 200 µm from the optic nerve head in the superior retina, shows that the ONL in treated retinas (17.47 ± 3.96 µm, *n* = 5) are significantly thicker than the control retinas (7.07 ± 1.12 µm, *n* = 5; *p* < 0.001, Student’s *t* test; Fig. 3*D*).

### Protection of cone photoreceptors by MANF

**Figure 4.**
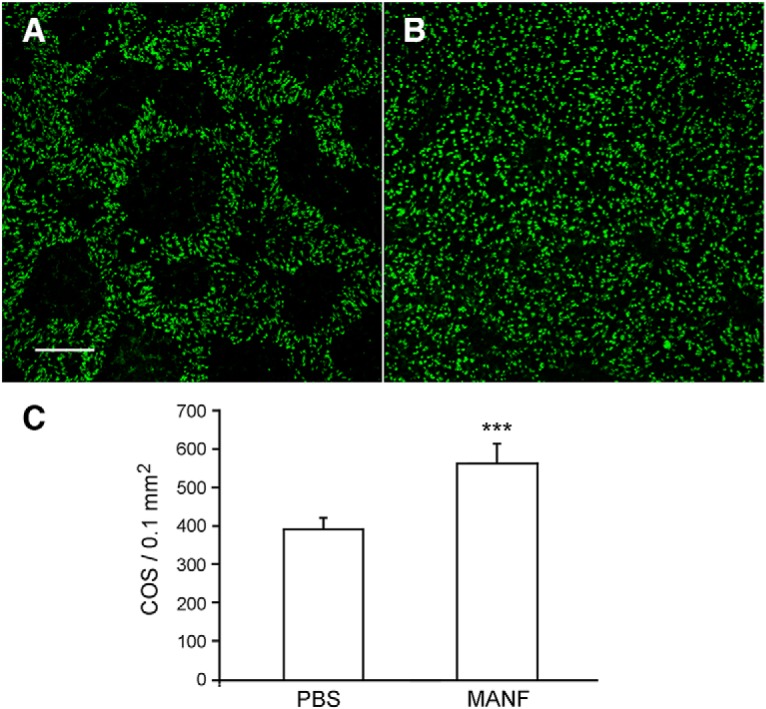
Protection of cone photoreceptors by MANF. The right eyes of S334ter-3 rats were treated with 6 µg (in 3 µl of PBS) of MANF by intravitreal injection, and the left eyes were injected with of PBS at PD20. Eyes were harvested 10 d after injection (PD30). Retinas were stained with PNA to identify cone outer segments. In the control retinas treated with PBS, many PNA-negative patches are present (***A***). In MANF-treated retinas, PNA-negative patches were very small and in many areas are not present (***B***). Quantitative analysis of PNA-positive cells in the superior retinas are significantly more in MANF-treated eyes (569.5 ± 46.5, *n* = 6) than in PBS-treated eyes (398.7 ± 25.4, *n* = 6; ***C***, triple asterisks indicate *p* < 0.001, Student’s *t* test). Scale bar: 100 µm.

The significant protective effect of rhMANF on rod photoreceptors encouraged us to examine the potential protective effect of rhMANF on cone photoreceptors. Cone photoreceptors in the S334ter-3 rats undergo significant secondary cone degeneration, characterized by loss of COSs in numerous PNA-negative patches throughout the retina (Li et al., 2010). We injected rhMANF (6 µg in 3 µl of PBS) intravitreally to the right eyes of S334ter-3 rats at PD20 when rod degeneration is mostly complete, and secondary cone degeneration is already significant (Li et al., 2010). The left eyes were injected with PBS as controls. Retinas were harvested at PD30 and stained with PNA. Many PNA-negative patches are present in the control retinas (Fig. 4*A*). In MANF-treated retinas, the PNA-negative areas are very small and in many cases are not present (Fig. 4*B*). Quantitative analysis showed that PNA-positive cells are significantly more in rhMANF-treated retinas (569.5 ± 46.5, *n* = 6) than in PBS-treated retinas (398.7 ± 25.4, *n* = 6; *p* < 0.001, Student’s *t* test; Fig.4*C*). These results indicate that MANF is also a potent neurotrophic factor for cone photoreceptors.

### ERG preservation by rhMANF treatment

**Figure 5.**
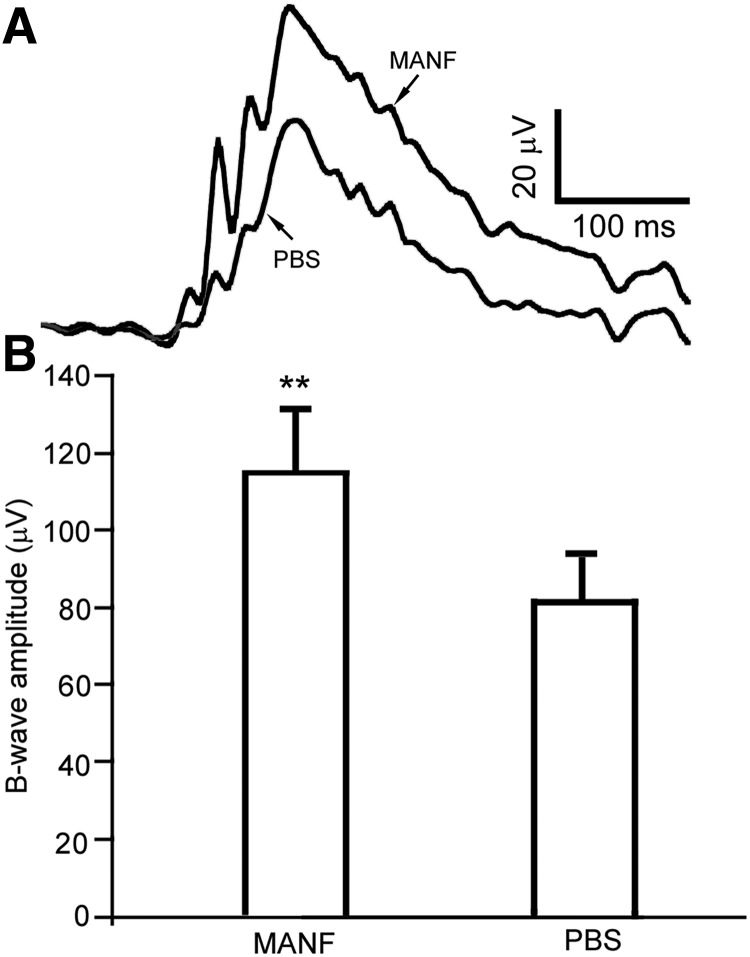
Preservation of ERG by MANF. The right eyes of *rd* 10 mice were treated with 2 µg (in 1 µl of PBS) of MANF by intravitreal injection at PD18. The left eyes were treated with PBS. ERGs were recorded from both eyes simultaneously 10 d later at PD28. Scotopic b-waves evoked by white light flashes of −0.4 log cd-s/m^2^ were reliably recorded but the a-waves were undetectable (***A***). Significantly higher b-wave amplitudes were recorded from MANF-treated eyes, with an average amplitude of 115 ± 16.5 μV (*n* = 5), as compared with PBS-treated eyes whose average amplitude is 82.4 ± 10.7 μV (*n* = 5; ***B***, double asterisks indicate *p* = 0.0017, Student’s *t* test).

We used the *rd10* mice, which carry a missense mutation in the *Pde6b* gene, to investigate the potential capacity of MANF to preserve the function of photoreceptors. The rod ERGs are suppressed in the *rd10* mice but measurable (Chang et al., 2002). In the ERG experiment, the left eyes were intravitreally injected with 2 µg of MANF (in 1 µl of PBS) at PD18, and the right eyes were injected with 1 µl of PBS to as controls. Full field ERGs were recorded from both eyes simultaneously at PD28. Scotopic ERG b-wave was reliably evoked by white light flashes of -0.4 log cd-s/m^2^, although the a-waves were undetectable (Fig. 5*A*). The average amplitude of b-wave from rhMANF-treated eyes is 115 ± 16.5 μV (mean ± SD, *n* = 5), significantly higher than the average amplitude of the b-wave from PBS treated control eyes of 82.4 ± 10.7 μV (*n* = 5; *p* = 0.017, Student’s *t* test; Fig. 5*B*).

## Discussion

We have investigated the spatial and temporal expression patterns of MANF in the retina, and the neuroprotective potential of MANF on photoreceptors. MANF is expressed in the retina at high levels during postnatal development. As the retina matures, the expression declines to lower levels. This temporal expression pattern in the retina is similar to the expression pattern of MANF in the cerebral cortex (Wang et al., 2014), suggesting the importance of MANF during the postnatal development and maturation of the retina and the brain. The extensive colocalization of MANF and GS immunoactivities shows that Müller cells are the major MANF-expressing cells and the major source of MANF in the retina.

The potency of MANF as a neurotrophic factor for photoreceptors is highlighted by the significant protection of photoreceptors. The rapid photoreceptor degeneration in the S334ter-3 rats (Liu et al., 1999) favors experiments to test purified protein factors, which could be degraded rapidly after intravitreal injection. Therefore this model ensures that only neurotrophic factors with enough potency could yield positive results. On the other hand, false negative results could occur when a factor with lower potency is tested. We used the S334ter-3 rats in our previous studies on photoreceptor protection by neurotrophic factors in the interleukin-6 family of neurotrophic cytokines, including CNTF, cardiotrophin-1 (CT-1), and oncostatin M (OsM); (Tao et al., 2002; Song et al., 2003; Xia et al., 2011).

In addition, rhMANF protects the function of photoreceptors, as indicated by the significantly preservation of ERGs in the *rd10* mice. The *rd10* mouse carries the p.R560C mutation in the *Pde6b* gene that leads to suppressed rod ERG and photoreceptor degeneration (Chang et al., 2002, 2007). The relatively late onset and milder degeneration in the *rd10* mouse makes it a preferred model for pre-clinical studies of retinal functions, including gene therapy trials (Pang et al., 2008, 2011), long-term neurotrophic factor study (Ohnaka et al., 2012), and small molecular compound treatment trials (Phillips et al., 2008; Kang et al., 2016).

MANF, also known as arginine-rich, mutated in early stage of tumors (Armet), is originally identified as a secreted protein in the culture medium of rat mesencephalic type 1 astrocytes that promotes survival of rat embryonic dopaminergic neurons (Petrova et al., 2003). It has no significant homology to any known neurotrophic factors and thus MANF is regarded as a novel neurotrophic factor, the founding member of a new family of neurotrophic factors (Lindholm and Saarma, 2010). A second member of the family, cerebral dopamine neurotrophic factor (CDNF), was identified a few years later (Lindholm et al., 2007). MANF is expressed in many areas in the CNS, including many brain regions and the spinal cord (Lindholm et al., 2008; Wang et al., 2014). It promotes survival of rat embryonic dopaminergic neurons *in vitro* (Petrova et al., 2003) and nigrostriatal dopaminergic neurons from 6-hydroxydopamine-induced degeneration *in vivo* (Voutilainen et al., 2009). Intracerebral injection of purified MANF reduces the volume of infarction and improves behavior recovery in a rat stroke model (Airavaara et al., 2009).

In addition to the CNS, MANF is found in the liver, the salivary gland, and the testis (Lindholm et al., 2008), suggesting that MANF also functions outside the CNS. Genetic ablation of the MANF gene in mouse results in progressive postnatal reduction of β-cell mass and severe diabetes (Lindahl et al., 2014), indicating that MANF is required for pancreatic β-cell proliferation and survival. A human patient with a mutation in the MANF gene has been reported to suffer from type-2 diabetes mellitus, hypothyroidism, primary hypogonadism, short stature, mild intellectual disability, obesity, deafness, high myopia, microcephaly, and partial alopecia (Yavarna et al., 2015), highlighting the roles MANF in many organs.

MANF is reported to interact with immune cells, and the interaction is shown to promote the integration of transplanted photoreceptor precursors, and to protect photoreceptors from light damage (Neves et al., 2016). However, the neurotrophic activity of MANF is independent of its interaction with immune cells, as it was originally identified in the conditional medium of VMCL1 astrocytes to protect dopaminergic neurons in an *in vitro* ventral mesencephalic neuroprotective assay (VMN assay) with no immune cells present (Panchision et al., 1998). The VMN assay was also used in the subsequent purification of MANF (Petrova et al., 2003). It is unlikely that the significant photoreceptor protection by recombinant MANF shown in the present study was resulted from interaction with immune cells. We recently observed interaction of MANF with Müller in the retina *in vivo*, and similar interaction was observed in cultured Müller cells *in vitro* with no immune cells present (R. Wen and Y. Li, unpublished observations).

The mechanism underlying the neurotropic effects of MANF is not clearly understood, and the putative receptor on the cell surface to interact with secreted MANF remains unknown (Voutilainen et al., 2015; Lindahl et al., 2017). MANF was reported to induce PKC phosphorylation in PC12 cells *in vitro* and cerebellum Purkinje cells *in vivo* (Yang et al., 2014). Structurally, MANF has two domains, the N-domain (amino-domain) and the C-domain (carboxyl-domain), which are believed to have distinct functions (Parkash et al., 2009; Lindahl et al., 2017). The N-domain is homologous to saposin-like proteins and is believed to have the extracellular neurotrophic activity. The C-domain is homologous to SAF-A/B, Acinus and PIAS (SAP) protein superfamily (Parkash et al., 2009; Lindahl et al., 2017).

MANF C-domain has been studied in details. It has a RTDL sequence for ER retention and is thought to function in ER stress response (Parkash et al., 2009; Lindahl et al., 2017). In addition, the CKGC motif in the C-domain, which forms the cysteine bridge, is involved in neuroprotection. Furthermore, the SAP domain is capable of inhibiting BAX-mediated apoptosis (Parkash et al., 2009; Liu et al., 2015; Mätlik et al., 2015; Lindahl et al., 2017). The C-domain is neuroprotective when expressed intracellularly (Hellman et al., 2011). Whether it can exert its neuroprotective activity when applied extracellularly remains to be studied. The N-domain is not well studied, and little is known about its functional role in neuroprotection.

In summary, we have shown that MANF is a retinal native protein expressed at high levels in early postnatal development. Müller cells are the major MANF-expressing cells, and neurons in the inner retina also express MANF. Intravitreal injection of rhMANF significantly protects both rod and cone photoreceptors, demonstrating that MANF is a potent neurotrophic factor for photoreceptors. These results provide experimental evidence to consider MANF as a neuroprotective agent for photoreceptor degenerative disorders, including RP and age-related macular degeneration. Further studies to understand the function of MANF N-domain could shed light on the extracellular neurotrophic activity of MANF. Identifying the cell surface receptors of MANF could lead to a better understanding of the molecular mechanism(s) that mediate MANF neurotrophic activity.

## Synthesis

Reviewing Editor: Julie Andersen, Buck Institute Decisions are customarily a result of the Reviewing Editor and the peer reviewers coming together and discussing their recommendations until a consensus is reached. When revisions are invited, a fact-based synthesis statement explaining their decision and outlining what is needed to prepare a revision will be listed below. Note: If this manuscript was transferred from JNeurosci and a decision was made to accept the manuscript without peer review, a brief statement to this effect will instead be what is listed below.

Both reviewers think the paper is very investing but both raised some concerns that should be addressed before the ms can be considered for publication. I summarize their comments below:

Reviewer 1:

n this manuscript, Lu et al. focused on a novel neurotrophic factor MANF (mesencephalic astrocyte-derived neurotrophic factor). MANF was originally identified as a secreted protein in the culture medium of rat mesencephalic type 1 astrocytes and protects nigrostriatal dopaminergic neurons from degeneration. Here, the authors detected the expression of MANF in the retina. During postnatal (P) development, they found the expression of MANF in retina was at high levels from P1 to P16 (peaked at P10, returning to low levels after P25). Through immunostaining, they also found MANF was widely distributed in the retina. Not only colocalized with GS+ cells (Müller cells), MANF was also distributed in the inner nuclear layer (INL), in ganglion cell layer (GCL), and in retinal pigment epithelial (RPE) cells. These results indicate that MANF was a retinal native protein. To test if MANF has a potential protective role on retinal degeneration, the authors adopted two rodent models of photoreceptor degeneration: S334ter-3 rats and rd10 mice. The authors expressed and purified His-tagged recombinant human MANF (rhMANF), and injected rhMANF intravitreally to the eyes of the rodent models. They found rhMANF injection increased the ONL (outer nuclear layer, where photoreceptor cell bodies localized) thickness and robustly decreased PNA (peanut agglutinin, which binds to cone outer segments) - negative areas in S334ter-3 rats. In rd10 mice, rhMANF supplementation rescued the suppressed ERG (electroretinograms). The authors concluded that MANF is a native protein in retina and is a potent neurotrophic factor for photoreceptor protection.

The paper has high translational value. Unfortunately, while this study was in progress, a protective role of MANF in retina degeneration rodent model was reported before (Neves, J., et al. 2016. Science 353, aaf3646). Expression of MANF in retina was shown in a recent article (Gao, F.J., et al. 2016. Front Hum Neurosci 10, 686). This current study extended previous studies and provide further and necessary evidence for MANF's potential as therapeutic intervention of retina degeneration using two additional/different retinal degeneration models. A novel finding of this paper is that MANF's photoreceptor protection effect is independent of its interaction with immune cells. The paper could be improved by addressing the following concerns.

More markers should be used for double immunostaining, especially when MANF is also detected in other layers. Besides, higher power images for colocalization should be presented.

For protection experiments using MANF, authors need to add a positive control, such as well-established CNTF, to compare the protection effects. In addition, different dosages of MANF should be used, rather than a single one.

His-tagged rhMANF was produced as a bacterial protein. Reference or evidence is needed that it is ‘active’.

In Fig. 3 and 4, anti-recoverin staining may be helpful. The ONL has both cones and rods. Which one is altered? Similar concerns are applicable to rd10 mice.

rhMANF injection appeared to increase ONL thickness. True? Can MANF reduce the apoptosis of photoreceptors? TUNEL staining should be included.

The mechanism underlying the protection effects of MANF should be explored. More evidence is necessary to exclude the role of immune cells.

Color and position of bars for quantitation should be consistent.

rhMANF and MANF were used interchangeably.

Reviewer 2:

In this study, the authors investigated the expression and role of the neurotrophic factor MANF in the neuroprotection of retina. They examined the expression and localization of MANF in the rat retina using Western blot analysis and immunostaining. They found that MANF is expressed in Müller cells, retinal ganglion cells, neurons in the inner nuclear layer neurons, and pigment epithelial cells, in an age-dependent manner. Intraocular injection of recombinant human MANF significantly reduced the death of rods and cones in rats carrying the rhodopsin S334ter mutation, and preserved ERG b-wave in the rd10 mice. Therefore, they claimed that MANF is a native protein in the retina and is a potent neurotrophic factor for photoreceptor protection.

Overall, this study addressed an important question regarding the neuroprotection during retinal degeneration. The experimental results are very interesting and potentially important and useful for the development of treatment strategy of retinal degeneration. However, the manuscript could potentially be enhanced significantly by addressing the following comments/suggestions.

1. Page 3: The statement of significance simply repeated the abstract. It should emphasize the ‘significance’ of the study.

2. Page 9, lines 200-202: It was stated “MANF immunoreactivity was also detected in neurons in the INL, in retinal ganglion cells in the GCL, and the RPE cells (Fig. 2B, D)”. However, no direct evidence was presented to demonstrate the MANF immunoreactivity overlaps with cell-specific staining of those cells.

3. Page 10, lines 211-214: Recombinant human MANF was injected intravitreally at PD9, and the eyes were collected at PD20 for histological analysis. Therefore, the protective effects were examined after a short-term period. However, it is not clear how long this protective effect will last. It will be much helpful to examine the treated eyes after a longer time period.

4. Page 10, lines 216-219 and lines 230-232: The results were compared between treated eyes and untreated degenerative eyes. It would be very helpful to demonstrate how significant the treatment of MANF on the protection of cells if the treated eyes were compared with normal, non-degenerative eyes.

5. Page 11, lines 240-241: It seems that only one light intensity was used to measure the scotopic ERG. It should also examine if photopic ERG is affected because the cone degeneration is also affected by the treatment.

6. Page 11, lines 241-244: Similar to the histological analysis, the ERG recording was performed at a short time after the treatment. So it is not clear how long the functional protection will last.

7. It is not clear why the histological analysis was conducted on S334ter-3 rats but the ERG recording was performed on rd10 mice. It would be more convincing/consistent if both the histological and functional studies were conducted on the same species.

8. Pages 11-12, second paragraph in Discussion: CNTF is one of the well-studied neurotrophic factors for neuroprotection and the authors have made significant contribution to the study of CNTF (page 5, first paragraph). As the authors state: “The extent of photoreceptor protection by MANF in the S334ter-3 rats is similar to, if not more than, neurotrophic factors in the interleukin-6 family of neurotrophic cytokines, including CNTF, CT-1 (cardiotrophin-1), and OsM (oncostatin M) (Tao et al., 2002; Song et al., 2003; Xia et al., 2011).” The authors should present more detailed quantitative comparison between MANF and other neurotrophic factors to support their statement.

9. Page 13, line 302-304: The mechanism underlying the neurotropic effects of MANF is not clearly understood, and the putative receptor interacts with secreted MANF remains unknown. It would be much stronger if any mechanistic study were conducted in this study.
